# Quantification and Comparison of Different Biofilm Components from Methicillin-Susceptible *Staphylococcus aureus* Treated with Tranexamic Acid Using an In Vitro Model

**DOI:** 10.3390/microorganisms13081874

**Published:** 2025-08-11

**Authors:** Marta Díaz-Navarro, Antonio Benjumea, Andrés Visedo, Patricia Muñoz, Javier Vaquero, Francisco Chana, María Guembe

**Affiliations:** 1 Department of Clinical Microbiology and Infectious Diseases, Hospital General Universitario Gregorio Marañón, 28009 Madrid, Spain; maartadn@gmail.com (M.D.-N.); andresvsg777@gmail.com (A.V.); pmunoz@hggm.es (P.M.); 2 Instituto de Investigación Sanitaria Gregorio Marañón, 28009 Madrid, Spain; vaquerocot@gmail.com; 3 Department of Orthopaedic Surgery and Traumatology, Hospital General Universitario Gregorio Marañón, 28009 Madrid, Spain; antonio.benjumea.carrasco@gmail.com (A.B.); chanaphd@gmail.com (F.C.); 4 CIBER Enfermedades Respiratorias-CIBERES (CB06/06/0058), 28029 Madrid, Spain; 5 School of Medicine, Universidad Complutense de Madrid, 28040 Madrid, Spain

**Keywords:** biofilm, *Staphylococcus aureus*, staining, CLSM, tranexamic acid, extracellular matrix, polysaccharides, proteins, DNA

## Abstract

As we previously demonstrated that tranexamic acid (TXA), an antifibrinolytic, showed an antibacterial effect alone and in combination with vancomycin and gentamicin, we now wanted to analyze its own efficacy using new, different fluorescent staining reagents that target different components of the biofilm matrix and compare which one quantifies biofilm reduction better. A 10^8^ cfu/mL suspension of the *Staphylococcus aureus* (ATCC29213) strain was placed into the wells of a 24-multiwell plate covered with glass slides coated with 10% poly-L-lysine under agitation for 24 h at 37 °C. After 3 washes with PBS, wells were treated with either TXA 10 mg/mL or sterile water and incubated for 24 h at 37 °C. After three washes with PBS, the density area of the following biofilm components was calculated using confocal laser scanning microscopy: extracellular proteins (Sypro Ruby), α-extracellular polysaccharides (ConA-Alexa fluor 633), α or β-extracellular polysaccharides (GS-II-Alexa fluor 488), bacterial DNA (PI), and eDNA (TOTO^®^-1). We observed a statistically significant reduction in the occupied area by all components of the *S. aureus* biofilm (*p* < 0.001) after TXA 10 mg/mL treatment, compared to the positive control. All biofilm components’ reduction percentages reached ≥90.0%. We demonstrated that TXA reduced both bacteria and extracellular matrix components of *S. aureus* biofilm by using five different stain reagents, with all being equally valid for quantification.

## 1. Introduction

Bacterial biofilms on medical devices are structured communities of microorganisms embedded in a protective extracellular matrix that adhere to device surfaces such as prostheses, developing periprosthetic joint infection (PJI) [1]. These biofilms exhibit increased resistance to antibiotics and host immune responses, making infections difficult to treat and often leading to chronic or recurrent conditions. Their formation is facilitated by bacterial communication systems like quorum sensing [2] and is a major contributor to hospital-acquired infections. The presence of biofilms on medical devices not only compromises patient safety but also increases morbidity, treatment costs, and the need for device removal or replacement, highlighting their critical importance in clinical settings [3,4]. Therefore, it is needed to carry out preventive strategies to avoid the appearance of bacterial biofilms.

Tranexamic acid (TXA) is an antifibrinolytic agent widely used in orthopedic surgery to reduce perioperative blood loss and minimize the need for blood transfusions [5]. The most common doses are 10–15 mg/kg intravenously and 1–3 g intra-articularly, depending on the type and complexity of the surgical procedure. Its primary mechanism of action involves the reversible blockade of lysine-binding sites on plasminogen molecules, thereby inhibiting its activation to plasmin, the enzyme responsible for breaking down fibrin clots. By stabilizing the fibrin matrix, TXA prevents premature clot degradation and promotes hemostasis during and after surgery. In orthopedic procedures, such as total joint arthroplasty or spinal surgery, where significant blood loss is common, TXA has proven effective in minimizing bleeding without significantly increasing the risk of thromboembolic complications when used appropriately. Therefore, we previously assessed whether it could have an antibacterial effect on planktonic and sessile cells [6,7,8,9,10], demonstrating that, despite that the effect was strain-dependent, it showed not only an antibacterial but also a synergistic effect with antibiotics such as vancomycin and gentamicin [11,12,13].

However, most of our findings were based on in vitro and in vivo models that assessed only specific biofilm properties, such as metabolic activity or qualitative aspects of the extracellular matrix and sessile cells by scanning electron microscopy [6,7,12,13]. Therefore, since confocal laser scanning microscopy (CLSM) is being increasingly used and new dye reagents are designed to target specific compounds of the biofilm matrix [14,15,16,17], we decided to expand our analysis. Thus, in the present study, we aimed to validate our anti-biofilm effect of TXA against *S. aureus* using a panel of different staining reagents that stain different biofilm components and to compare which one quantifies biofilm reduction better.

## 2. Method

The study was carried out in the microbiology laboratory of a tertiary teaching Institution in Madrid, Spain.

A few colonies from a fresh 24 h culture of methicillin-susceptible *S. aureus* (ATCC29213) isolated from enriched culture medium were placed in 20 mL of Tryptic Soy Broth (TSB) with subsequent incubation under orbital shaking for 24 h at 37 °C. The suspension was diluted in the same broth to obtain a 10^8^ cfu/mL bacterial solution that was inoculated into the wells of 24-multiwell plates. Previously, 24 glass slides pre-coated with 10% poly-L-lysine for 24 h at 37 °C under agitation (150 rpm) were added to each well to promote bacterial adhesion. After this time, the glass slides were washed with Phosphate-Buffered Saline (PBS) and treated with either Tranexamic acid (TXA) 10 mg/mL or sterile water in the case of the positive control (as it was the solvent used for TXA preparation). The plates were incubated for 24 h at 37 °C and subsequently washed with PBS and allowed to dry. Thus, 24 h-formed biofilms on the surface of glass slides were used to quantify the area density of biofilm components. To do this, biofilms were treated with 0.5% Triton-X 100 and 4% formaldehyde solution as a detergent to disrupt and fix them, respectively, so that biofilms could then be processed with the corresponding fluorescent reagent and obtain an image of 24 h biofilm growth accumulation on surfaces. The staining reagents were the following: Sypro Ruby (binds to extracellular proteins of biofilms), Concanavalin A conjugated with Alexa fluor 633 dye (for labelling α-extracellular polysaccharides), *Griffonia simplicifolia* Lectin (GS-II) conjugated with Alexa fluor 488 dye (to identify α or β-extracellular polysaccharides), propidium iodide (PI) (to label bacterial DNA), and Thiazole Orange Homodimer (TOhD, TOTO^®^-1) (binds to extracellular DNA [eDNA]). An application time for each staining reagent was used according to the manufacturer’s recommendations. Stained biofilm samples were examined using a Leica TCS SPE confocal fluorescence microscope (Leica Geosystems AG, Heerbrugg, Switzerland). The biofilm depth was measured at 4 µm intervals from the bottom of the biofilm along 80 µm with a 10× objective. Finally, images were processed using FIJI (Image J) software (National Institute of Health, Bethesda, MD). Data on bacterial density was estimated at the stacks’ maximum z-projections. Three fields per sample were obtained, and biofilm components’ density values were calculated as the percentage of occupied area by each of them.

We only tested 10 mg/mL of TXA because, as we demonstrated in previous studies, it was enough to observe the antibacterial effect without reaching a toxic concentration [8,9,10].

### Data Analysis

Quantitative variables are expressed as mean ± standard deviation (SD), and the non-parametric Mann–Whitney test was applied to analyze statistical differences between the treatment group and controls. Significance was set at *p* < 0.05.

All tests were performed using SPSS Statistics for Windows, v.21.0 (IBM Corp, Armonk, New York, NY, USA).

Data are stored in repository C.0001228 of the ISCIII.

## 3. Results

Table 1 presents the occupied area of some components of biofilms produced by methicillin-susceptible *Staphylococcus aureus* (MSSA) in the two study groups: one treated with 10 mg/mL tranexamic acid (TXA) and a control group (+C). The biofilm components analyzed include extracellular proteins, α-extracellular polysaccharides, α-β-N-acetylglucosamine, bacterial DNA, and extracellular DNA (eDNA), each stained with their specific fluorescent dyes. Across all components, the TXA-treated group exhibited significantly lower occupied area percentages compared to the control group. For example, extracellular proteins showed a mean area of 0.15 ± 0.01% in the TXA group versus 17.58 ± 1.22% in controls, and similar trends were observed for polysaccharides (1.69 ± 0.69% vs. 16.34 ± 4.71%), α-β-N-acetylglucosamine (0.57 ± 0.28% vs. 16.77 ± 1.36%), bacterial DNA (1.60 ± 0.81% vs. 16.55 ± 13.42%), and eDNA (0.07 ± 0.02% vs. 12.43 ± 6.23%). All differences were statistically significant with *p* < 0.001, indicating that TXA markedly inhibits MSSA biofilm formation by reducing the accumulation of key structural and genetic components.

**Figure 1:**
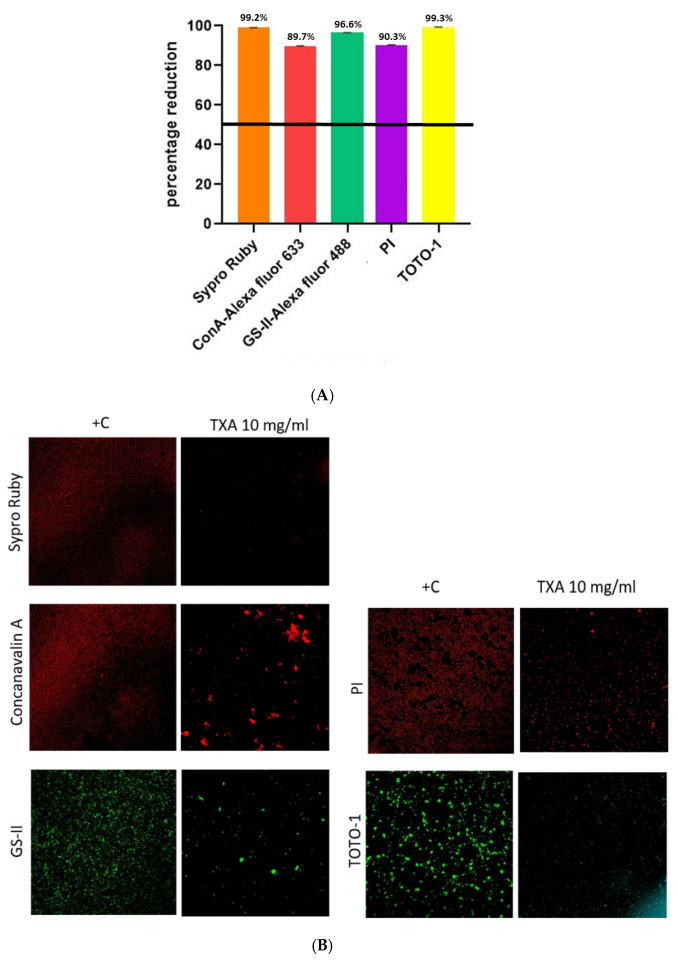
Analysis of methicillin-susceptible *Staphylococcus aureus* biofilm. (**A**). Mean percentage reduction of density according to each component after TXA 10 mg/mL treatment. (**B**). CLSM images of methicillin-susceptible *Staphylococcus aureus* biofilm treated or not with TXA 10 mg/mL using 5 different dyes. TXA, tranexamic acid; +C, positive control; ConA-Alexa, Concanavalin A conjugated with Alexa fluor 633; GS-II, *Griffonia simplicifolia* Lectin (GS-II) conjugated with Alexa fluor 488; PI, propidium iodide; TOTO-1, Thiazole Orange Homodimer (TOhD, TOTO^®^-1).

In Figure 1A, the mean (±SD) percentage reductions in biofilm components are represented as follows: extracellular proteins (Sypro Ruby), 99.2% (±0.1); α-extracellular polysaccharides (Con-A), 89.7% (±0.3); α or β-extracellular polysaccharides (GS-II), 96.6% (±0.1); bacterial DNA (PI), 90.3% (±0.1); and eDNA (TOTO^®^-1), 99.3% (±0.2). A representative CLSM image of *S. aureus* biofilm stained by each reagent according to TXA treatment vs. positive control is shown in Figure 1B, which demonstrates statistically significant reductions in the number of viable cells associated with all analyzed components of the biofilm matrix, as indicated by differential staining with specific reagents. Marked decreases were observed in samples stained for extracellular proteins (Sypro Ruby), α-extracellular polysaccharides (ConA–Alexa Fluor 633), α-β-N-acetylglucosamine (GS-II–Alexa Fluor 488), bacterial DNA (PI), and extracellular DNA (TOTO-1). These findings suggest that the treatment condition substantially impairs biofilm viability and structural integrity, as evidenced by diminished cellular and matrix-associated signal intensities across all staining modalities.

## 4. Discussion

One of the main factors associated with the development and persistence of periprosthetic joint infection (PJI) is bacterial adhesion to the implant surface; thus, once adhered, bacteria can form a biofilm. Biofilms create a protective environment for bacteria, which makes it difficult to eradicate the infection due to the increased resistance to both the immune system and antimicrobial treatment conferred by this structure [3,4].

Recent research has explored a variety of strategies to solve biofilm-related infections. Among these, tranexamic acid (TXA) has been shown to reduce postoperative complications in prosthetic surgery due to its dual action: both by an anti-fibrinolytic indirect effect and by an antibacterial direct effect [6,7,8,9,10]. Regarding other alternative strategies, there are few prophylactic therapies that have been successfully applied to address the management of intra- and postoperative infections. In addition to TXA, there has been recent research on the use of antiseptic agents in prophylaxis in the context of PJI [18] and the use of local antibiotics such as vancomycin powder [19]. However, despite promising results, there is no absolute standardized protocol or specific institutional guidelines for their use in routine clinical practice either.

Moreover, our study group also demonstrated that not only did it have an antibacterial effect but also a synergistic effect with other antibiotics [11,12,13]. Despite the fact that our findings were reliable enough to confirm their effect, we also consider that other additional laboratory techniques could help to validate these results and, in the process, take advantage of this to explore the ability of different types of dyes designed to bind specific components to biofilm matrix targets. Our results allowed us to confirm that all stains were useful to assess the effect of TXA on *Staphylococcus aureus* biofilms more accurately in terms of quantification of the biofilm matrix percentage reduction in TXA-treated *S. aureus* biofilms vs. non-treated biofilms (≥90% reduction).

Regarding the laboratory methods used to mimic biofilm-related infections, there are many both in vitro and in vivo models commonly used [20,21]. Although in vitro models differ from in vivo conditions (not fully replicating in vivo conditions), they offer a controlled and reproducible environment and represent an important support to evaluate the level of biofilm formation and the direct effect of any drug on the components of the biofilm matrix, which is actually responsible for preventing current antimicrobial therapy from penetrating the structure to be effective. Moreover, specific staining to evaluate the number of different biofilm characteristics combined with confocal microscopy also offers a promising tool for analyzing biofilm structure and composition. As it was previously suggested in the literature [16,17,18,19], there are useful fluorescent dye reagents capable of selectively staining specific biofilm compounds, such as extracellular proteins, polysaccharides, bacterial DNA, or eDNA, allowing for detailed analysis of their response to treatment. In our study, we have successfully validated and demonstrated that all of these reagents are reliable and useful to assess the efficacy of TXA against *S. aureus* biofilms. Particularly relevant is the decrease in N-acetyl glucosamine, an extracellular polysaccharide that, when self-secreted, promotes bacterial adhesion in the biofilm formation process and contributes to the development of a lower immune response [22]. Furthermore, we did not use IP as a marker of cell death, but rather to geolocate bacteria within the biofilm. Therefore, based on the area occupied by the IP marker, we can deduce that TXA could have an effect not only on the biofilm matrix but also on the bacteria that comprise it. With all this, it seems that our results could be extrapolated to any other compound and to any other bacterial biofilm, making the biofilm staining-based technique a useful tool that could be standardized in microbiology laboratories to facilitate the screening of new anti-biofilm agents.

Nevertheless, questions remain regarding the exact mechanisms by which TXA exerts its anti-biofilm activity. It is still unknown whether an indirect mechanism of action could explain the effect of TXA on *S. aureus* biofilm by the alteration of the pH of the extracellular medium. It has been shown that an increase in the pH of the local environment causes an alteration in the association of cell matrix proteins, making their adhesion to the medium difficult and altering the formation of biofilm, being an important secondary mechanism that contributes to its anti-biofilm effect [23].

As a limitation of the study, there are no specific and universal techniques for staining the different matrix components of all bacterial or fungal biofilms. This is due to the complex composition of the matrix, which depends on the genus and even the species, generating wide diversity and complexity in biofilm matrices. Thus, the staining reagents used were individually selected for the species investigated (*S. aureus*) and may not be extrapolated to be used with other bacterial or fungal biofilms [24]. Furthermore, as we only used PI and it penetrates only through damaged cell membranes, labeling only dead cells that we previously drilled holes into and fixed Triton X-100 and paraformaldehyde, it might be more appropriate to also stain them with SYTO9, a fluorescent dye that binds to the DNA of all bacteria present in the samples since it is capable of penetrating the membrane, thus indirectly acting as a live cell dye. This would give us a more realistic view of what is happening within the biofilm matrix.

## 5. Conclusions

In conclusion, we demonstrated that tranexamic acid (TXA) reduced both bacteria and extracellular matrix components of *Staphylococcus aureus* biofilms by using five different fluorescent stain reagents, with all being equally valid for quantification. These findings support TXA’s potential as an anti-biofilm agent through diverse analyses.

## Figures and Tables

**Table 1:** Components’ biofilm density from methicillin-susceptible *S. aureus* in both study groups.

Component	Dye	Mean ± SD Occupied Area (%)		*p*
		10 mg/mL TXA	+C	
Extracellular proteins	Sypro Ruby	0.15 ± 0.01	17.58 ± 1.22	<0.001
α-extracellular polysaccharides	ConA-Alexa fluor 633	1.69 ± 0.69	16.34 ± 4.71	<0.001
α-β-N-acetil glucosamine	GS-II-Alexa fluor 488	0.57 ± 0.28	16.77 ± 1.36	<0.001
Bacterial DNA	PI	1.60 ± 0.81	16.55 ± 13.42	<0.001
eDNA	TOTO-1	0.07 ± 0.02	12.43 ± 6.23	<0.001

TXA, tranexamic acid; +C, positive control; ConA-Alexa, Concanavalin A conjugated with Alexa fluor 633; GS-II, *Griffonia simplicifolia* Lectin (GS-II) conjugated with Alexa fluor 488; PI, propidium iodide; TOTO-1, Thiazole Orange Homodimer (TOhD, TOTO^®^-1).

## Data Availability

The original contributions presented in this study are included in the article. Further inquiries can be directed to the corresponding author.
